# Addressing the cost of infractions in the online literature and databases

**DOI:** 10.1371/journal.pone.0188761

**Published:** 2017-11-30

**Authors:** Rodney J. Dilley, Oliver G. Ash

**Affiliations:** 1 Ear Science Institute Australia and Ear Sciences Centre, Division of Surgery, School of Medicine, University of Western Australia, Nedlands, Western Australia, Australia; 2 University of Notre Dame, Fremantle, Western Australia, Australia; University of Illinois-Chicago, UNITED STATES

## Abstract

Myocardial infarction sometimes appears misspelt as myocardial infraction in the cardiovascular research literature. With accurate citation of literature contributions important to advancing the field and its contributors, in this study we aimed to document the frequency and explore the causes and impact of this error specific to the cardiology literature.

Literature databases (PubMed, Scopus, Web of Science, WIPO, Google Scholar, Google) were searched to identify the rate of myocardial infraction errors and found an error rate between 0.2% and 1.6%, with substantial differences between search tools used. A Scopus search was used to show changes in errors over time, differences between journals and by specific authors. Myocardial infraction occurred at increasing annual rates over time and at higher rates than other errors. Increasing error rates were associated with increased volume of searchable material rather than quality of the literature. Transcription from article to database is a common source of error and some databases have higher rates of these errors. Simple measures to avoid and to correct these errors in the literature and the databases are also discussed.

## Introduction

**Fig 1:**
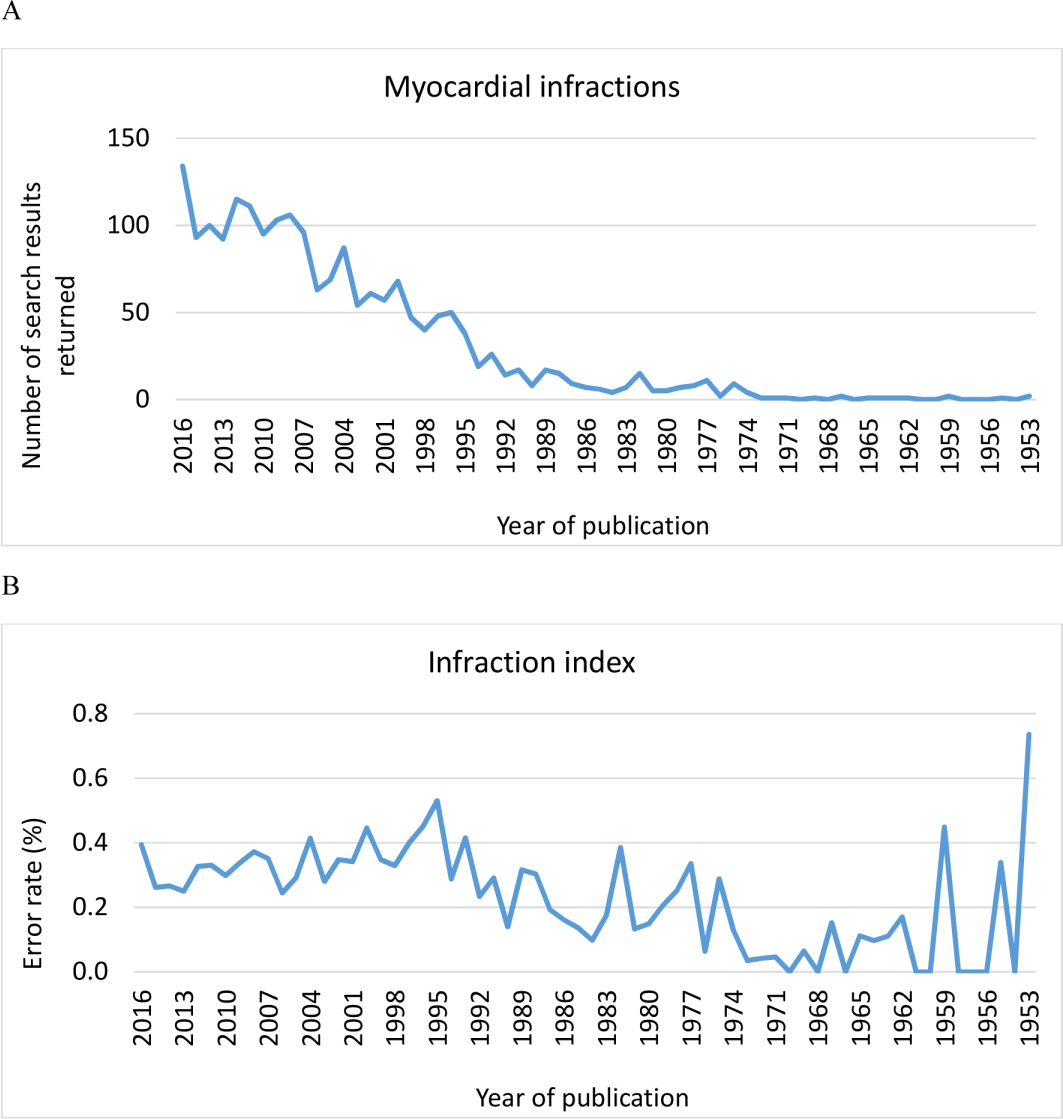
Yearly rates of myocardial infraction and infraction index from 1953 to 2016. (A) Number of papers retrieved for each year from a Scopus “all fields” search for “myocardial infraction”. (Accessed January 4, 2017). (B) The infraction index over the same time frame, calculated as a percentage of errors from the same search for “myocardial infarction”. (Accessed January 4, 2017).

“Myocardial infraction” is a word combination not detected by most spellcheck software, so in the cardiovascular literature it appears often as a mistyped “myocardial infarction”. Despite the resources available throughout the publishing chain to correct misspellings, myocardial infraction has occurred year on year at increasing rates since the 1970s (Fig 1A). Recent infractions appear in the highest level cardiovascular specialist journals such as Circulation [1] and JACC [2], in more general science journals such as PLoS One [3] and also rarely in general medical journals such as Lancet [4]. PubMed* finds 378 myocardial infraction hits since 1953. Web of Science (501) and Scopus (2108) find more and Google, the ultimate blunt search weapon with the broadest search scope, finds a massive 134,000. At 12,500,000 hits for the intended search “myocardial infarction”, an “infraction index” can be derived (100 x hits for myocardial infraction / (hits for myocardial infarction +myocardial infraction)). Comparing search engines for infraction index produces the following rank order: Google (1.06%) > Google Scholar (0.75%) > Scopus (0.31%) > Web of Science (0.19%) > PubMed (0.17%). A patent search (WIPO) produced 1.6%. These differences between search engines likely derive from the scope of their searches and their accuracy. Google searches available information from the entire World Wide Web, including all text and images from webpages or documents; other databases search a narrower set of documents and some less comprehensively in each document. Thus the net cast by Google and Google Scholar is very broad; including grey literature, duplications and weakly associated entries [5], capturing both more instances of infraction and a higher error rate in less extensively edited sources. These differences require consideration by researchers with the obvious trade-off between accuracy and breadth of a search.

Using a Scopus search, annual rates of myocardial infraction have increased 20-fold since 1985 through 2016 (Fig 1A), but infraction index increased only two-fold during this time (Fig 1B), suggesting that increase in infractions is due more to increased volume of papers being published in this area than to more errors. A further inference is that quality has been largely maintained through this period of substantial technology disruption, with now universal adoption of personal word processor software (and spell checkers) and transition to online publishing of many journals.

Of the many infraction errors published in literature and searched in databases, myocardial infraction may be most relevant to cardiologists. A “cancre” (cancer) index is nearly 50 fold lower than myocardial infraction at 0.01% and an “intestinal infraction” index is 0.07%, but with only 1 hit, does not have the same impact. A “cerebral infraction” index of 0.23% (with a more appreciable 192 hits on Scopus) also occurs in the stroke literature. Interestingly, Freiberg’s infraction, a necrosis of the metatarsal head, is spelled as Freiberg’s infarction in 17.4% (37 hits) of Scopus entries, and may be a product of inappropriate spelling correction as well as an unusual mixed usage pattern for the two terms.

### Sources of the infractions

Journals vary in their infraction index (Table 1). A Scopus search of prominent cardiology journals (those with >5000 hits for infarction), ranks Circulation (0.23) and American Journal of Cardiology (0.33) with the least infractions, European Heart Journal (0.47), American Heart Journal (0.47) and JACC (0.48) had the most. The author list from our Scopus search is headed by some of the biggest names in the field. Robert M Califf has a powerful track record (1435 authorships in Scopus since 1978) and also the most myocardial infractions (14, at 0.97). Interestingly, PubMed reports 0 infraction hits for the same author. Closer inspection of the Scopus data reveals that all Califf’s infractions are database transcription errors and do not appear in the original journal articles, so the good doctor’s writing credentials are in the clear. In this case the search engine creates the error. With no monitoring of quality of database transcription outside of the database providers themselves, it is up to the searcher to beware. Certainly, Califf and others may wish to encourage Scopus to scrutinise their database more closely.

**Table 1:** The top ten journals ranked by infraction index, based on numbers of papers returning at least one “myocardial infraction” error in a Scopus search (1953 to 2017).

Journal	Myocardial infraction	Myocardial infarction	Infraction index (%)
Journal of Psychosomatic Research	12	773	1.53
Japanese Circulation Journal	23	1607	1.41
Annales De Cardiologie Et D Angeiologie	9	919	0.97
Acta Cardiologica	9	956	0.93
Revista Espanola De Cardiologia	20	2284	0.87
Journal Of Cardiovascular Medicine	8	935	0.85
European Journal Of Preventive Cardiology	9	1053	0.85
Ugeskrift For Laeger	7	842	0.82
Journal Of Nuclear Cardiology	10	1285	0.77
Diabetic Medicine	5	654	0.76

Other examples of transcription errors can be ascribed to the journals. A well cited 1993 paper in JACC [6] from the EAMI trial appears in Scopus and Scholar as “Long-term physical training and left ventricular remodelling after anterior myocardial infraction: Results of the excercise in anterior myocardial infraction (EAMI) trial.” These title errors do not appear in the original article PDF, PubMed or Web of Science searches, yet the journals internal search database does contain the errors and these are transmitted to Scholar and Scopus.

Editorial staff hold the final responsibility for typographical errors in print, and as with most human endeavours, they are not perfect. Even with the substantial software resources used in various iterations of the preparation of a journal article by authors, reviewers, editors, copyeditors, database entry and search text entry, mistyping to form an English word such as infraction is difficult to detect. This might be exacerbated as authors who do not have English as their native language publish more articles or as non-native speakers are involved in the editorial and production process. It might be further influenced by changes in the publishing industry as disruptive technologies arise and time pressures on the publishing process increase. Vigilance will be required to maintain quality.

There may be rare deliberate uses of myocardial infraction. In the only example we could find, Karen Wright used it as a clever wordplay to title her article on (mis)interpretation of cholesterol evidence [7]. The rest are apparent errors. Eminent cardiologist Charles K. Friedberg erroneously used it to title his symposium summary in 1972 [8], the year of his untimely death. Interestingly, Scopus and PubMed have corrected both the Friedberg and Wright instances to myocardial infarction in their databases, even though both articles clearly show infraction in their original titles, and one of them intentionally. An exact word match PubMed search (”myocardial infraction”) does not retrieve either article. In contrast, D.M. Lawrie wrote “myocardial infraction” in the Lancet in 1969 [9], which is faithfully reproduced on the Lancet website and online search engines. These errors passed through the editorial process to be published but not necessarily cited. Which is the correct way to cite such errors? The AMA style manual does not provide any specific guidance and convention suggests that citations should appear verbatim from the original.

### Consequences of the error

Aside from irritating the pedants among us, such errors may be truly significant for a works impact in the field by producing missed retrievals in searches. Potentially valuable insight into an area is lost to the researcher or there may be unnecessary duplication of work. These consequences may be less significant in the clinic than published dosage errors or protocol errors. However, lost citations can have personal impact for the researchers on grant funding and academic research appointments, which depend in part on these metrics. Lack of awareness of existing patents, for example, can be costly in time and money. From the examples given above, Wright’s paper has no citations recorded for Google Scholar, Friedberg’s paper has 45 citations under “Infraction” and 16 different citations for the same paper under “Infarction”. “Myocardial infraction” and “infarction” may also appear in the same paper, reducing the risk of lost search retrievals. Searching just in article titles reveals myocardial infraction appears alone 99% of the time, but in “all fields” searches only 23% are lone infractions at risk of lost citations. Intuitively, when the infraction appears in the title the impact on citations may be higher. The highest cited paper with infraction in the title [6] has nearly 10 times less citations (155 vs 1535) than the highest cited paper where infraction appears elsewhere in the article and is ranked as only the 57^th^ highest cited infraction paper overall. The impact of these and other lost citations can’t be easily determined.

### How to avoid the error

Intuitively this appears to be a manageable source of error. The substantial spellcheck resources available to authors, reviewers, editors, copyeditors, and for search text entry and database entry catch most, but mistyping to form incorrect combinations of English words is ultimately difficult to detect. During searching, PubMed and others point to alternative searches (i.e. myocardial infarction) at the top of their first results page, however the pointer does not appear on subsequent search results pages and is not present in secondary searches from software such as Endnote. To avoid the error during searching, use of MeSH terms will ensure correct spelling and avoiding “title only” searches will reduce the risk further. Vigilant checking for infraction before submission and publication of myocardial infarction papers is required, and in text entry for databases. A simple solution might be for journals publishing in this field to routinely check for infractions in submitted manuscripts during other routine (e.g. plagiarism) checks.

### Correcting citation errors

Where infraction appears in the cited article title, accurate citing of referenced articles will ensure correct attribution to the authors, however automated spell checking in search engines may identify and correct the error and inadvertently result in lost citations if the correction is published.

For published errors, PubMed has recently revised their error correction protocol and all changes must now be made through the publisher of the affected journal. Previously a conservative approach required formal application and erratum statements to be published in the journal before corrections could appear in PubMed. Transcription errors at the database may now be corrected directly by the publisher using the PubMed Data Management System. The protocol for submission of corrections at each publisher will now dictate the timeliness of those PubMed corrections identified by authors. Despite this improved new system, there does not appear to be an industry standard error correction protocol for publishers or for databases. With increasing numbers of infractions perhaps it is time to consider an approach to resolve them systematically. Certainly, there would be benefits for authors like Wright, Friedberg and Lawrie, whose infractions may otherwise be consigned to history.

*All searches were performed using Scopus [all fields] for “myocardial infraction” on September 22, 2017, except where indicated. The widest search settings were used (e.g. all fields, all dates, all languages) with no additional filters for each database.

## Supporting information

S1 File Scopus search raw data for analysis of myocardial infraction and infarction. Data spreadsheet is provided showing original data exported from Scopus searches and for calculation of Infraction Index.
(XLSX)
